# HMDB 5.0: the Human Metabolome Database for 2022

**DOI:** 10.1093/nar/gkab1062

**Published:** 2021-11-19

**Authors:** David S Wishart, AnChi Guo, Eponine Oler, Fei Wang, Afia Anjum, Harrison Peters, Raynard Dizon, Zinat Sayeeda, Siyang Tian, Brian L Lee, Mark Berjanskii, Robert Mah, Mai Yamamoto, Juan Jovel, Claudia Torres-Calzada, Mickel Hiebert-Giesbrecht, Vicki W Lui, Dorna Varshavi, Dorsa Varshavi, Dana Allen, David Arndt, Nitya Khetarpal, Aadhavya Sivakumaran, Karxena Harford, Selena Sanford, Kristen Yee, Xuan Cao, Zachary Budinski, Jaanus Liigand, Lun Zhang, Jiamin Zheng, Rupasri Mandal, Naama Karu, Maija Dambrova, Helgi B Schiöth, Russell Greiner, Vasuk Gautam

**Affiliations:** Department of Biological Sciences, University of Alberta, Edmonton, AB T6G 2E9, Canada; Department of Computing Science, University of Alberta, Edmonton, AB T6G 2E8, Canada; Department of Laboratory Medicine and Pathology, University of Alberta, Edmonton, AB T6G 2B7, Canada; Faculty of Pharmacy and Pharmaceutical Sciences, University of Alberta, Edmonton, AB T6G 2H7, Canada; Department of Biological Sciences, University of Alberta, Edmonton, AB T6G 2E9, Canada; Department of Biological Sciences, University of Alberta, Edmonton, AB T6G 2E9, Canada; Department of Computing Science, University of Alberta, Edmonton, AB T6G 2E8, Canada; Department of Computing Science, University of Alberta, Edmonton, AB T6G 2E8, Canada; Department of Biological Sciences, University of Alberta, Edmonton, AB T6G 2E9, Canada; Department of Biological Sciences, University of Alberta, Edmonton, AB T6G 2E9, Canada; Department of Computing Science, University of Alberta, Edmonton, AB T6G 2E8, Canada; Department of Biological Sciences, University of Alberta, Edmonton, AB T6G 2E9, Canada; Department of Biological Sciences, University of Alberta, Edmonton, AB T6G 2E9, Canada; Department of Biological Sciences, University of Alberta, Edmonton, AB T6G 2E9, Canada; Department of Biological Sciences, University of Alberta, Edmonton, AB T6G 2E9, Canada; Department of Biological Sciences, University of Alberta, Edmonton, AB T6G 2E9, Canada; Department of Biological Sciences, University of Alberta, Edmonton, AB T6G 2E9, Canada; Department of Biological Sciences, University of Alberta, Edmonton, AB T6G 2E9, Canada; Department of Biological Sciences, University of Alberta, Edmonton, AB T6G 2E9, Canada; Department of Biological Sciences, University of Alberta, Edmonton, AB T6G 2E9, Canada; Department of Biological Sciences, University of Alberta, Edmonton, AB T6G 2E9, Canada; Department of Biological Sciences, University of Alberta, Edmonton, AB T6G 2E9, Canada; Department of Biological Sciences, University of Alberta, Edmonton, AB T6G 2E9, Canada; Department of Biological Sciences, University of Alberta, Edmonton, AB T6G 2E9, Canada; Department of Biological Sciences, University of Alberta, Edmonton, AB T6G 2E9, Canada; Department of Biological Sciences, University of Alberta, Edmonton, AB T6G 2E9, Canada; Department of Biological Sciences, University of Alberta, Edmonton, AB T6G 2E9, Canada; Department of Biological Sciences, University of Alberta, Edmonton, AB T6G 2E9, Canada; Department of Biological Sciences, University of Alberta, Edmonton, AB T6G 2E9, Canada; Department of Biological Sciences, University of Alberta, Edmonton, AB T6G 2E9, Canada; Department of Biological Sciences, University of Alberta, Edmonton, AB T6G 2E9, Canada; Department of Biological Sciences, University of Alberta, Edmonton, AB T6G 2E9, Canada; Department of Biological Sciences, University of Alberta, Edmonton, AB T6G 2E9, Canada; Department of Biological Sciences, University of Alberta, Edmonton, AB T6G 2E9, Canada; Department of Biological Sciences, University of Alberta, Edmonton, AB T6G 2E9, Canada; Leiden Academic Centre for Drug Research LACDR/Analytical Biosciences, Leiden University, Leiden, Netherlands; Laboratory of Pharmaceutical Pharmacology, Latvian Institute of Organic Synthesis, Riga, Latvia; Section of Functional Pharmacology, Department of Neuroscience, Uppsala University, Uppsala, Sweden; Institute for Translational Medicine and Biotechnology, Sechenov First Moscow State Medical University, Moscow, Russia; Department of Computing Science, University of Alberta, Edmonton, AB T6G 2E8, Canada; Department of Biological Sciences, University of Alberta, Edmonton, AB T6G 2E9, Canada

## Abstract

The Human Metabolome Database or HMDB (https://hmdb.ca) has been providing comprehensive reference information about human metabolites and their associated biological, physiological and chemical properties since 2007. Over the past 15 years, the HMDB has grown and evolved significantly to meet the needs of the metabolomics community and respond to continuing changes in internet and computing technology. This year's update, HMDB 5.0, brings a number of important improvements and upgrades to the database. These should make the HMDB more useful and more appealing to a larger cross-section of users. In particular, these improvements include: (i) a significant increase in the number of metabolite entries (from 114 100 to 217 920 compounds); (ii) enhancements to the quality and depth of metabolite descriptions; (iii) the addition of new structure, spectral and pathway visualization tools; (iv) the inclusion of many new and much more accurately predicted spectral data sets, including predicted NMR spectra, more accurately predicted MS spectra, predicted retention indices and predicted collision cross section data and (v) enhancements to the HMDB’s search functions to facilitate better compound identification. Many other minor improvements and updates to the content, the interface, and general performance of the HMDB website have also been made. Overall, we believe these upgrades and updates should greatly enhance the HMDB’s ease of use and its potential applications not only in human metabolomics but also in exposomics, lipidomics, nutritional science, biochemistry and clinical chemistry.

## INTRODUCTION

The Human Metabolome Database (HMDB) is the world's largest and most comprehensive, organism-specific metabolomic database. It contains richly annotated, carefully cross-checked, extensively referenced information about all currently known human metabolites. This includes information contained in HMDB’s ‘MetaboCard’ on their chemical structures, names or identifiers, detailed textual descriptions, references, chemical taxonomy, biological roles, physiological concentrations, tissue/biofluid locations, disease associations, genetic associations, chemical and enzymatic reactions, metabolic pathways and referential MS/MS (tandem mass spectrometry), GC–MS (gas chromatography mass spectrometry), and NMR (nuclear magnetic resonance) spectra. The HMDB supports a wide range of interactive web queries that allow metabolomic researchers to identify and annotate human (and other mammalian) metabolomic data through text, structure, mass or spectral matching. Unlike general metabolism or metabolic pathway databases such as KEGG (1), Reactome (2) and the Cyc databases (3) or general spectral databases such as the BioMagResBank (4), Metlin (5) or MassBank [https://massbank.eu/MassBank/], the HMDB is not simply an archival database of compounds or spectra. Rather it is a colorfully illustrated, extensively annotated online encyclopedia covering almost everything that is known about human metabolites and human metabolism.

Since its first release in 2007 (6), the HMDB has gone through extensive development and improvement to meet the changing needs of the metabolomics community. These changes have also been driven to keep pace with new metabolite discoveries and new insights in metabolism, to stay current with advancing metabolomic technologies, and to adapt to changes in modern web design and data delivery technologies. Over the past 15 years the changes to the HMDB have been quite remarkable. The first release of the HMDB (HMDB 1.0) contained limited biological, physiological and physico-chemical data on just 2180 human metabolites (6). HMDB 2.0, which was released in 2009, included more spectral data and much more literature-derived physiological and biochemical data for 6408 human metabolites (7). HMDB 3.0, which appeared in 2013, contained a total of 40 153 human metabolites (8). This third release greatly expanded the HMDB’s spectral reference library, added metabolic pathway data and modernized the HMDB user interface. HMDB 4.0, which was published in 2018, contained at total of 114 100 compounds (9). This version massively increased the number of NMR, MS/MS and GC–MS reference spectra and the number of illustrated metabolic pathways. It also added new data on metabolic reactions, pharmacometabolomic data, metabolite-SNP associations and introduced the ClassyFire (10) chemical taxonomy.

For this year's release of HMDB (version 5.0), the HMDB curation team has implemented a number of very significant and noteworthy improvements to the database. In particular, the HMDB 5.0 now has 217 920 annotated metabolite entries, as well as another 1 581 537 unannotated derivatized metabolite entries for GC–MS. As part of a large-scale, multi-year database update, the HMDB 5.0 has also significantly improved quality and depth of metabolite descriptions, with thousands of metabolite descriptions being manually or semi-manually rewritten, corrected and expanded. The HMDB 5.0 now includes a new Chemical Functional Ontology (ChemFOnt) which provides a more machine-readable route to extract metabolite functions and origins. In addition, many new, far more powerful and far more interactive structure, spectral and pathway visualization tools have been added to HMDB 5.0. Likewise, 9 445 375 highly quality predicted spectral data sets and other experimental ‘observables’ have been added to the database, including 312 980 predicted ^1^H and ^13^C NMR spectra, 1 752 677 more predicted GC–MS spectra, 1 440 324 predicted LC–MS/MS spectra, 5 067 714 predicted retention indices and 871 680 predicted collision cross section values. Finally, significant enhancements to the HMDB’s search functions have been added to facilitate better and more accurate compound identification. More details describing these improvements are given under the following five subsections: (i) New Metabolite Entries; (ii) Improved Metabolite Descriptions; (iii) New Visualization Tools; (iv) New Spectral Data and (v) Improved Search Functions.

## NEW METABOLITE ENTRIES

One of the most significant challenges facing metabolomics researchers concerns the annotation or identification of m/z features in MS spectra obtained from metabolomics studies. In many cases, the number of *m*/*z* features in untargeted MS-based human metabolomics studies that can be confidently identified is typically <2% (11). Even among targeted metabolomics studies, it is rare to identify >900 human metabolites (12), which is <1% of the known human metabolome. This strongly suggests that both the metabolite coverage and the MS spectral coverage in the HMDB (and other databases) has been inadequate or incomplete.

To address these issues, a concerted effort was undertaken by the HMDB curation team to increase the HMDB’s metabolite coverage. From 2018 to 2020 a continuous scan of the literature as well as a more detailed historical review of published metabolomics and exposomics studies was conducted. This led to the addition of another 1476 metabolites to the database. Beginning in 2021 a more focused effort was undertaken to expand HMDB’s coverage of oxidized lipids (i.e. lipids with oxidized acyl chains) (13), additional cardiolipins, exposome or environmental compounds identified in human blood (14), acylcarnitines and their corresponding acylCoAs (15), as well as acylamides (16). We also included novel bile acid-amino-acid conjugates (17), food-derived compounds (https://foodb.ca), sulfated metabolites (18), other newly identified human metabolites, newly approved drugs and a number of microbially or gut-derived metabolites. These additions required careful review of dozens of papers and textbooks, of which only a few exemplar references are mentioned here. In total, 40 142 new oxidized lipids, 52 783 new cardiolipins, 14 929 blood exposome compounds, 2165 acylcarnitines and acylCoAs and 188 acylamides were added to the database. In addition, 65 bile acid amino-acid conjugates, 3168 food-derived compounds, 35 additional sulfated metabolites, 65 newly approved drugs, 19 new microbially derived compounds and 9 novel, experimentally identified metabolites were also added to the database. In total, 113 568 new compounds were appended to HMDB 5.0. In addition, another 9548 BioTransformer-predicted compounds as well as 323 disproven or erroneous compounds along with several duplicate entries were removed. As a result, HMDB 5.0 now has a total of 217 920 compounds. Over and above these annotated metabolites, the HMDB also maintains an unannotated collection of 1 581 537 derivatized compounds which correspond to TMS and TBDMS derivatized metabolites that could potentially be detectable via GC-MS methods. This derivatized compound collection is described in more detail later.

All of the exposome compounds identified in human blood, the bile amino-acid conjugates, the food-derived compounds, the sulfated metabolites, other newly identified human metabolites, newly approved drugs and microbially or gut-derived metabolites are classified in the HMDB as either ‘detected but not quantified’ or ‘detected and quantified’ metabolites. This classification, which has been in place in the HMDB since 2013, simply means there is solid experimental evidence and literature data supporting the metabolite's existence and/or quantification. On the other hand, the vast majority of the newly added lipids and lipid/acyl derivatives, are classified in the HMDB as ‘expected but not quantified’ compounds. This category, which has also been in place since 2013, includes those metabolites that are expected to exist based on biochemistry, enzymology or known constituents (i.e. acyl chains) found in the human body. Evidence for their structure and existence is ascertained based on an extensive literature review by the HMDB curation team, along with a detailed analysis of known constituents in human samples and putative identifications reported from human metabolomic studies of various biofluids and tissues. In all cases, specific literature references are provided to support the existence of these newly added compounds.

Each newly added metabolite in the HMDB 5.0 has gone through HMDB’s comprehensive data update process. In particular, every metabolite is given an accession number and a variety of in-house programs and commercial software tools are run to collect, calculate, generate or predict data covering up to 130 data fields for each compound's MetaboCard. Many of the resulting data updates (or selected samples) are manually reviewed by members of the curation team to ensure consistency and accuracy. Details on the data sources, curation protocols, data harvesting and description-writing software (ChemoSummarizer and Data Wrangler), prediction software, data management system, and quality assurance criteria for the HMDB have been described previously (8,9).

## IMPROVED METABOLITE DESCRIPTIONS

A particular strength of the HMDB, and one of the main reasons for its popularity within the metabolomics community, is its rich collection of metabolite descriptions. Every compound in the HMDB has a detailed textual description ranging from 50 to 500 words that describes the compound, what it does or where it's located in the body or in the cell. In addition, many metabolites in the HMDB have additional information regarding their occurrence in biofluids or tissues, their normal/abnormal concentrations, their disease associations, their MS and/or NMR spectra, their known pathways, their external database hyperlinks and their associated enzymes or transporters.

To update the HMDB 5.0, a concerted effort was made to manually research, rewrite and remediate compound descriptions for more than 800 well-known or disease-associated metabolites. This process required hundreds of hours of intensive literature research and writing. In addition, dozens of lesser-known metabolites with inadequate or incomplete descriptions were also manually researched and re-written. For those HMDB compounds that were modular in structure (i.e. lipids, bile acids and acyl derivatives), so-called ‘template descriptions’ were carefully written by hand and then computer programs were run to generate >200, 000 individualized or customized compound descriptions using these fill-in-the-blank templates. In addition, HMDB’s ChemoSummarizer (a program that has been used to auto-describe compounds in the HMDB since 2017) was modified and upgraded to incorporate more chemical/biological data, to extract data from DrugBank (19) and MarkerDB (20) and to generate more informative descriptions. Overall, these improvements to the quality and coverage of HMDB’s descriptions should greatly enhance the overall utility and reliability of the database.

In addition to these ‘human readable descriptions’, further development also continued with HMDB’s chemical functional ontology, called ChemFOnt (9), which is displayed under each MetaboCard's ‘Ontology’ field. ChemFOnt was first introduced in HMDB 4.0 as a hierarchically structured ontology that was both OWL (Web Ontology Language) and OBO (Open Biological Ontology) compliant. ChemFOnt was developed to help establish a chemical/biochemical ontology for the metabolomics community that could complement the better-known Gene Ontology or GO (21). GO is widely used in the proteomics/genomics community to assist with gene annotation and pathway analysis. The near-term goal for ChemFOnt has been to help automate and extend metabolite descriptions within the HMDB. The second near-term goal is to make HMDB’s compound descriptions more fully machine readable. The 2017 version of ChemFOnt covered four major functional categories (process, role, physiological effect and disposition) that were associated with 35 subcategories and 3150 descriptors or definitions. For HMDB 5.0, ChemFOnt has grown to include 247 subcategories, with a hierarchical structure of up to 6 nested categories and a total of 221 454 definitions. All categorical assertions or assignments in ChemFOnt have been restructured to have clear provenance with either a database or a literature reference. Furthermore, the quality and correctness of the ChemFOnt entries have been greatly improved over what was initially presented in HMDB 4.0. Every metabolite entry in HMDB 5.0 now has a hierarchically structured, fully hyperlinked ChemFOnt table where every descriptor definition can be accessed by mousing over the term of interest. Much more detailed disposition data about the origin (food, microbial, endogenous), originating species, biofluid and body site of many metabolites (especially food and microbial metabolites) is now provided through ChemFOnt. The data within ChemFOnt is still evolving and growing on a daily basis. This is because functional data is being continuously added to every HMDB entry through ongoing ‘background’ data mining and natural language processing activities. These background processes are being run and overseen by the HMDB curation team. Over the next year it is expected that nearly every ChemFOnt entry will contain significantly more human-readable and machine-readable information than what is available in standard HMDB compound descriptions.

In addition to these widespread improvements in HMDB’s compound descriptions, another major data update effort has been directed to expanding the amount of experimentally measured data in HMDB 5.0. This experimentally measured data includes more quantitative data on vitamin levels (normal and abnormal), extensive metabolite data on the human fecal metabolome (22), quantitative data on reference values for urinary metabolites in newborns (23) and substantially more quantitative data on the NIST (National Institute of Standards) human serum reference known as SRM-1950 (24). These updates are visible under the ‘Normal/Abnormal Concentration’ field in each MetaboCard. In total, more than 19 715 compound concentrations have been added, corrected or annotated. Likewise, significant numbers of experimental NMR and MS data for purified reference compounds have also been added. These include reference NMR spectra from ongoing activities within the Wishart laboratory (309 experimental NMR spectra for 218 compounds) as well as 37 589 experimental MS spectra made available from MassBank and other MS spectral providers. These updates are visible under the ‘Spectra’ field in each MetaboCard

## NEW VISUALIZATION TOOLS

**Figure 1.**
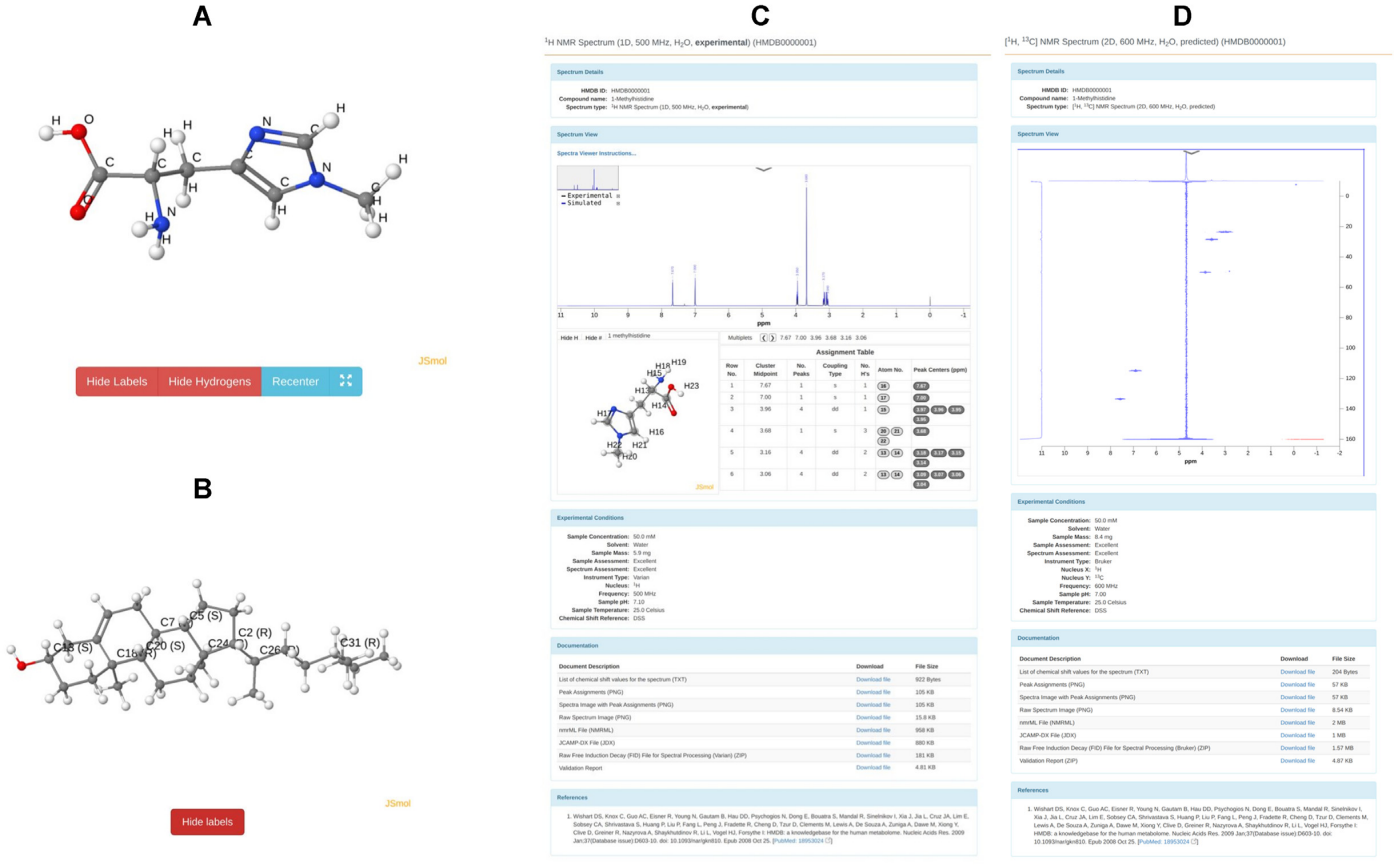
A screenshot montage of some of the new visualization features in HMDB 5.0. (**A**) an example of a 3D-rendered JSmol image of 1-methylhistidine as offered through HMDB’s ‘View in JSmol’ viewing option; (**B**) an example of the stereo-labeling (i.e. R/S) of Campasterol now offered through HMDB’s ‘Stereo view’ viewing option; (**C**) An example of a 1D ^1^H NMR spectrum of 1-methylhistidine as viewed through HMDB’s new JSpectraViewer (JSV); (**D**) and example of a 2D ^13^C–^1^H HSQC NMR of 1-methylhistidine as viewed through HMDB’s new JSpectraViewer.

Continuing improvements to JavaScript technology have meant that more sophisticated and more interactive visual displays are now possible on most modern web servers and web-based databases. In an effort to improve the quality of its structure visualization tools, HMDB 5.0 now includes several new tabs in the ‘Structure’ field of each MetaboCard. In addition to providing new tabs for ‘3D SDF’, ‘3D MOL’ and ‘PDB’ formatted files, users can now select a light blue tab under the thumbnail image, called ‘View in JSmol’, to visualize the 3D structure of the molecule via JSmol (25). This generates an interactive 3D display of the molecule (in a new window) that supports mouse-driven rotation and zooming of the molecule. Users may also select another light blue tab below the thumbnail structure called ‘View Stereo Labels’ to visualize the structure with the absolute configuration (R/S annotation) indicated in all chiral centres. Examples of these images are shown in Figure 1.

New spectral viewing options are also now available in HMDB 5.0. Each MetaboCard now has three spectral data field headers (orange or tan-colored bars) marked as (i) MS/MS Spectra; (ii) GC–MS Spectra and (iii) NMR Spectra. Clicking the ‘View Spectrum’ button tab takes users to the HMDB spectral viewing page (which differs slightly between NMR and MS spectra). This page displays all the spectral and associated metadata, including general MS or NMR spectral information, interactively viewable MS or NMR spectra, experimental acquisition data, downloadable documentation and/or spectral files and literature references. This page also provides hyperlinks on the right side to navigate through the page(s). Both NMR and MS spectra can be viewed through this ‘View Spectrum’ page via a locally developed JavaScript spectral viewer called JSpectraViewer or JSV (9). For predicted MS data, JSV allows users to mouse over each peak to interactively and see the predicted mass and fragment ion structure. The MS data for both experimental and predicted spectra are available and downloadable as lists of m/z values and intensities (in *.txt format) and in an mzML format.

For NMR data, JSV, is somewhat more sophisticated and now supports the display of both 1D and 2D NMR spectra (Figure 1). JSV displays NMR peak/chemical shift assignments both on the NMR spectrum and on the molecule itself, which is shown as a thumbnail image with numbered atoms and an assignment table. In the spectral view window JSV displays blue traces, which correspond to the predicted/simulated NMR spectra while the black traces correspond to the experimental NMR spectra. Only those entries with experimental NMR spectra will display both blue and black traces. Predicted or simulated NMR spectra only have blue traces. For experimental spectra, users can now toggle between the black (experimentally acquired spectrum) and the blue (simulated spectra). Blue traces will be very slightly different from black traces as the assignment process inherently leads to some information loss (including some couplings). JSV for NMR also supports interactive spectral zooming, moving, gridding, scaling and image saving/downloading. Interactive zooming, peak identification and peak picking are also supported by the 2D version of JSV. Each NMR spectrum of a pure compound (experimental or predicted) in the HMDB has downloadable information in the form of a set of peak lists (CSV format), peak assignments (CSV), spectral images (PNG), a spectral and/or assignment validation report and the actual or simulated NMR data in the form of nmrML (26) and JCAMP-DX files (27). If experimental data are available, the documentation section also provides native free-induction-decay (FID) or time-domain data in the original depositor format (Bruker, Varian, Agilent, JEOL).

**Figure 2.**
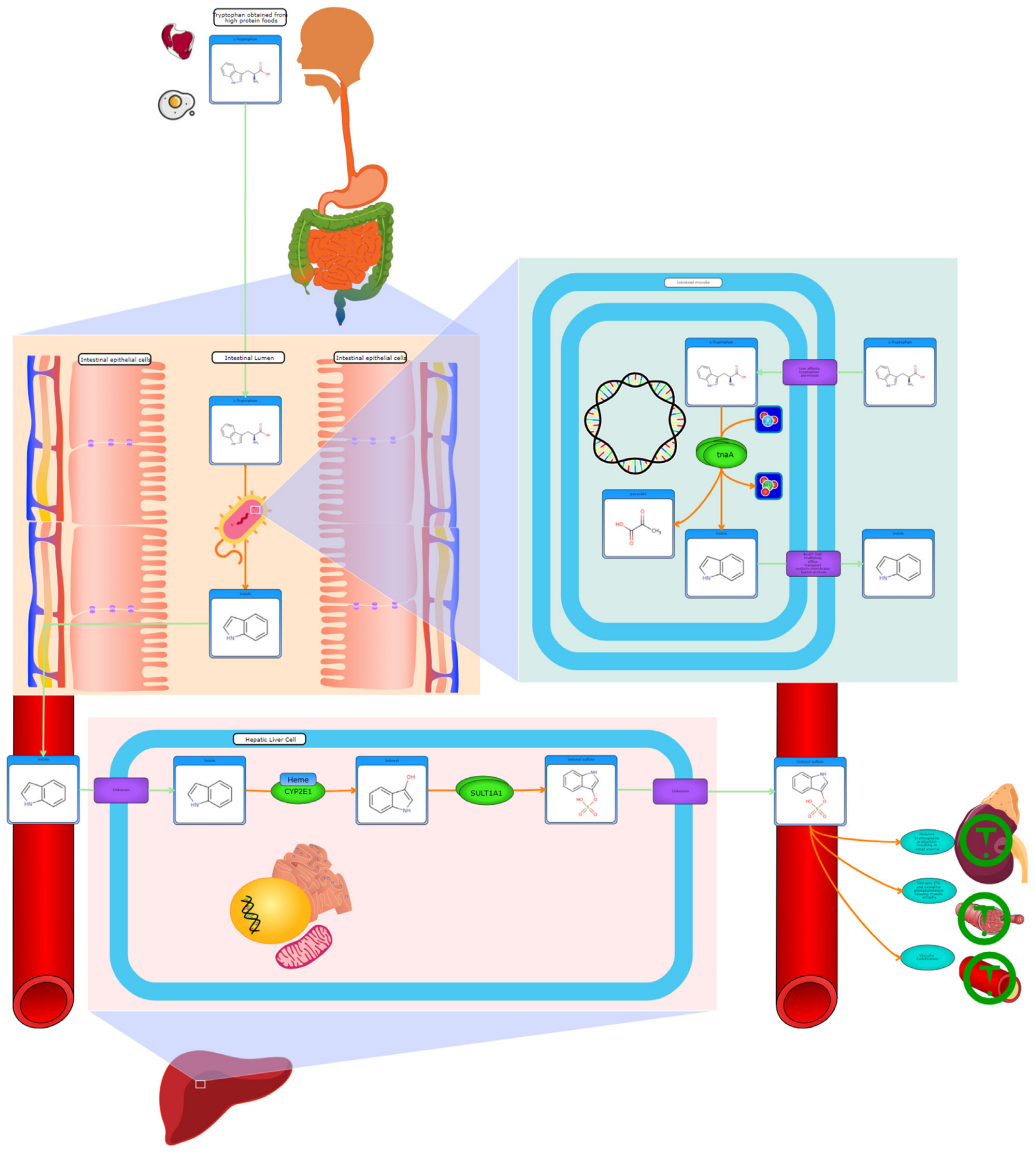
An example of an HMDB pathway (generated via the online pathway drawing tool PathWhiz) describing the mode of action and mechanism of formation of the uremic toxin known as indoxyl sulfate. This illustrates the breadth of molecular, subcellular, cellular and organ/tissue renderings as well as the breadth of physiological effect renderings that are now possible with PathWhiz and the PathBank pathways now linked to the HMDB. More than 100 000 pathways are now linked to metabolites in HMDB 5.0.

Further improvements in HMDB’s pathway visualization tools have also continued with HMDB 5.0. Pathways images created by PathWhiz (28) as part of the PathBank project (29) have become increasingly standardized, more fully annotated and more visually sophisticated relative to the pathway images released in HMDB 4.0. PathWhiz is an online pathway drawing server which has been used to populate PathBank, which is a dedicated metabolomics pathway database covering many model organisms. Hundreds of old or outdated PathBank pathways have been remediated and enhanced with improvements to the layouts, images, formatting and pathway descriptions/references. Many improvements in the images for subcellular structures, tissues and organs have also been made, allowing for much more advanced pathway processes to be illustrated. Similarly, a larger variety of action icons in the PathWhiz illustration palette are permitting sophisticated physiological processes and drug actions to be illustrated. An example of a uremic toxin pathway (illustrating the toxic action and effects of indoxyl sulfate) is shown in Figure 2. Alternate coloring schemes and alternative pathway layouts are also offered including a (default) colored, data rich pathway rendering, a black-and white rendering, and a simplified KEGG-like pathway rendering. All of the thumbnail PathBank pathway images link to full size interactive pathway views that can be saved and downloaded in static image formats (PNG and SVG) as well as in a variety of common data exchange formats such as SBML (systems biology mark-up language), BioPax and PathWhiz's own markup language (PWML). HMDB 5.0 now has 132 335 metabolite pathways covering 136 878 metabolites or xenobiotics and 2153 proteins.

## NEW SPECTRAL DATA

Key to the identification and annotation of metabolites for metabolomics researchers is the availability of spectral ‘observables’ linked to specific reference compounds. Comparing experimentally acquired observables to databases of reference compounds and reference observables allows metabolites to be identified. These observables may include parent ion masses, adduct masses, MS/MS spectra (at different collision energies), EI-MS spectra, ^1^H or ^13^C NMR spectra, collision cross section (CCS) data, retention indices and retention times. The HMDB 5.0 has continued to expand its collection of experimentally collected observables, including MS/MS, EI-MS and NMR spectra. However, it is clear that the number of new and metabolically relevant experimental MS and NMR spectra being deposited in public databases is rapidly diminishing. Likewise, the coverage offered by these experimentally measured observables is typically <5% (often much less) of the HMDB. It is because of this limited coverage that the HMDB curation team has placed increasing emphasis on generating accurately predicted observables. While experimentally collected observables are always preferrable over predicted observables, predictions have the advantage of offering complete or near-complete metabolome coverage.

For HMDB 5.0 significant resources were placed into developing or implementing: (i) more accurate MS/MS spectral predictions; (ii) accurate 1D ^1^H and ^13^C NMR spectral predictions; (iii) accurately predicted retention indices for analyzing GC–MS data and (iv) accurately predicted collision cross section (CCS) data for analyzing ion mobility spectroscopy (IMS) data.

The MS/MS predictions for HMDB 5.0 were performed by the latest version of the competitive fragment modeling tool for QTOF MS/MS spectral prediction, called CFM-ID version 4.0 (30). CFM-ID 4.0 was trained on a much larger data set and included much more sophisticated machine learning approaches to improve its handling of ring cleavages and molecular topology. It also incorporates hand-made fragmentation rules to handle lipids, polyphenols, acylcarnitines and other ‘modular’ molecules. As measured by a number of objective criteria, the performance of CFM-ID 4.0 is approximately 30% better than previous versions of CFM-ID (30). As a result, CFM-ID 4.0 was used to predict both the positive ion and negative ion mode QTOF MS/MS spectra of all 217 920 metabolites in the HMDB 5.0, at three different collision energies (10, 20 and 40 eV). This led to the generation of 1 440 324 MS/MS spectra (six spectra for each metabolite entry in HMDB 5.0), with several million predicted fragment labels—all of which have been rendered for interactive display in each HMDB MetaboCard by JSV. These MS/MS data have also been incorporated into HMDB’s new MS/MS search function. Note that Orbitrap MS spectra closely resemble QTOF MS/MS spectra (differing primarily in peak intensities but not in their peak positions) and that spectral matching with CFM-ID predicted QTOF MS/MS spectra against Orbitrap MS spectra often yields excellent results (30).

In addition to generating >1.4 million MS/MS spectral predictions to help improve MS-based compound identification, the HMDB curation team also generated 312 980 ^1^H and ^13^C NMR spectral predictions to help with NMR-based compound identification. The number of experimental NMR spectra available for metabolite identification in metabolomics has always been disappointingly low (<1000) and has increased only marginally in the past 10 years. Given the very limited compound coverage of reference metabolite NMR spectra, we decided to perform chemical shift and NMR spectral predictions on all water-soluble (predicted log *P* < 0 and –2 < log *S* < 0) metabolites in the HMDB. This threshold cutoff led to the selection of 15 649 molecules from the HMDB. Recent advances in NMR theory along with continuing innovations in computing techniques are allowing remarkably accurate NMR spectral simulations and NMR parameter predictions to be made for many small molecules (31–33). In particular, it is now quite routine to predict not only ^1^H and ^13^C shifts but also ^1^H and ^13^C NMR spectra from chemical structures with impressive accuracy (< 0.15 ppm RMSE for ^1^H shifts and <1.5 ppm RMSE for ^13^C shifts). The chemical shift predictors we employed use a combination of machine learning techniques and HOSE-code methods that nearly identical to the predictors available via NMRShiftDB (34). Empirically derived rules were used to predict first-order *J*-coupling constants and simple spin matrix calculations were used to generate the predicted 1D ^1^H and ^13^C NMR spectra at 10 different spectrometer frequencies (100 MHz to 1000 MHz for ^1^H and 25 MHz to 250 MHz for ^13^C). This led to the generation of 312 980 NMR spectra (20 spectra for each water-soluble metabolite entry in HMDB 5.0)—all of which have been rendered for interactive display in each HMDB MetaboCard by JSV. These NMR data have also been incorporated into HMDB’s new ‘NMR Search’ function, which is described later.

Retention indices (RI) are another useful set of observables that can be used to identify molecules. RIs are essentially adjusted retention times used in gas chromatography that allow nearly universal comparisons of retention times across GC platforms. RIs are closely related to a molecule's boiling point and are much more reproducibly measured in GC than retention times in liquid chromatography. Tens of thousands of retention indices for thousands of compounds (and their TMS or TBDMS derivatives) have been compiled in databases over the past 40 years. Experimentally measured retention indices can be used to help greatly narrow down possible candidates and are often used to assist in the identification of compounds by GC–MS. Important developments have recently occurred in the accurate (<2% RMSE) prediction of GC-MS retention indices that make use of sophisticated deep learning methods (35). The HMDB curation team adopted the same machine learning methods described by Qu *et al.* (35) and obtained essentially the same RI performance that was reported. Using a cutoff mass of 900 daltons (the upper mass limit for most GC-MS instruments), a total of 57 648 compounds were selected from the HMDB as being ‘GC–MS’ compatible. These compounds were then computationally derivatized with TMS and TBDMS to generate 1 581 537 derivatized structures. The RI predictor was then used to predict the retention indices for these 1.58 million derivative structures across three standard types of GC columns (semi-standard non-polar, standard non-polar and standard polar). This led to the generation of 4 744 611 predicted column-specific retention indices—all of which have been entered in the ‘Predicted Spectral Properties’ subsection (under the ‘Physical Properties’ field) of every eligible HMDB MetaboCard. These retention indices, the corresponding metabolite derivative structures and the CFM-ID predicted EI-MS spectra (36) for all 1 581 537 structures have also been incorporated into HMDB’s new ‘GC–MS Search’ function, which is described later.

The development of ion mobility spectroscopy (IMS) and the appearance of tandem IMS-MS systems has led to a growing interest in the metabolomics community in using IMS as a constraint to help with metabolite identification. Like GC separations, IMS separations are highly reproducible and far more consistent or predictable than LC separations. Furthermore, IMS retention values are related to the average collision cross section (CCS) of the molecule, which can be accurately predicted based on a compound's 3D structure. For HMDB 5.0 we have used a number of published CCS predictors, included MetCCS and DeepCCS (37–39) to generate the CCS values for all HMDB metabolites. Most of these CCS predictors report errors of <3–4%. Using these predictors, a total of 871 680 predicted CCS values have been added to the HMDB. All predicted CCS values been entered in the ‘Predicted Spectral Properties’ subsection (under the ‘Physical Properties’ field) of every HMDB MetaboCard. These CCS values have also been incorporated into HMDB’s new ‘LC–MS Search’ and ‘LC–MS/MS Search’ functions, which are described later.

## IMPROVED SEARCH FUNCTIONS

The addition of many new or newly predictable spectral observables (CCS, RI, NMR chemical shifts, etc.) also necessitated a substantial upgrade to the spectral search functions for HMDB 5.0. Furthermore, improvements in our spectral visualization program (JSV), also allowed us to undertake improvements in the graphical display of the spectral match output. Both the ‘LC–MS Search’ and ‘LC–MS/MS Search’ functions now support IMS data as an additional search constraint. Both have an option to input a CCS value with a default 5% tolerance. Users may choose any one of three specific CCS predictors or an averaged value of all three CCS predictors. If no CCS input value is provided, the search functions will still perform their regular MS or MS/MS searches without the CCS constraint. Matched compounds for HMDB’s new ‘LC–MS Search’ are ranked according to their m/z and CCS matches (using a combined weight of 90% for delta *m*/*z* and 10% for delta CCS). The output table from HMDB’s ‘LC–MS Search’ provides a browsable list that contains information on the matching compound names, the HMDB links, their m/z values, the CCS matches (if a CCS value was provided) and the overall score. In a similar manner, matched compounds for HMDB’s new ‘LC–MS/MS Search’ are ranked according to their spectral similarity and CCS matches. The output table from HMDB’s ‘LC–MS/MS Search’ provides a browsable list that contains information on the matching compound names, the HMDB links, their m/z values, spectral similarity, CCS similarity (if a CCS value was provided) and the overall score. Clicking on the ‘Show Spectrum’ produces a JSV mirror plot with the input spectrum shown at the top (in red) and the matching MS/MS spectrum shown at the bottom (in blue). Both ‘LC–MS Search’ and ‘LC-MS/MS Search’ have a ‘Load Example’ button to illustrate how these new search functions work.

HMDB’s new ‘GC–MS Search’ has now been modified to support RI data as an additional search constraint. Users may input an RI value with a default 3% tolerance. If the RI option is chosen, users must also choose any one of three types of GC columns as the RI values are specific to the column type (the default is the most popular column: semi-standard non-polar). Additionally, the type of chemical derivatization(s) used must also be provided. Users have the option to indicate no derivatization, TMS derivatization, TBDMS derivatization or combinations of the above. If no RI input value is provided, the GC-MS search function will still perform its regular EI-MS-only search without the RI constraint. Matched compounds for HMDB’s new ‘GC–MS Search’ are ranked according to their spectral similarity and RI similarity. The output table from HMDB’s ‘GC–MS Search’ provides a browsable list that contains information on the matching compound names of the underivatized parent compound, the HMDB links, the names of the derivatized compounds, their m/z values, spectral similarity, the RI matches (if an RI value was provided) and the overall score. Clicking on the ‘Show Spectrum’ button for any given compound produces a JSV mirror plot with the input spectrum shown at the top (in red) and the matching EI-MS spectrum shown at the bottom (in blue). As with other search functions, ‘GC–MS Search’ has a ‘Load Example’ button to illustrate how the new search function works.

HMDB’s new ‘NMR Search’ has been simplified and it now allows users to enter lists of ^1^H or ^13^C chemical shifts to search for spectral matches to experimental NMR spectra, predicted NMR spectra or both. Users must provide a chemical shift list (relative intensities are optional), select the nucleus (^1^H or ^13^C) of interest and choose a chemical shift tolerance (default of 0.2 ppm for ^1^H and 2.0 ppm for ^13^C) before pressing the ‘Search’ button. A typical query produces a browsable table of hits showing eight columns: the compound name, the HMDB ID (with a hyperlink tab), the structure, the chemical formula, the molecular weight (average and monoisotopic), the chemical shift Dice score (a measure of chemical shift matching), the fraction of peak matches and a colored ‘Show Spectrum’ button. Clicking on the ‘Show Spectrum’ produces a JSV mirror plot with the input NMR spectrum shown at the top (in red) and the matching NMR spectrum shown at the bottom (in blue). As with other search functions, ‘NMR Search’ has a ‘Load Example’ button to illustrate how the new search function works.

Overall, these improvements to HMDB’s search functions along with significant improvements to the quantity and quality of the underlying data should greatly enhance the performance and reliability of the HMDB as a ‘go-to’ resource for metabolite annotation. These improvements should also increase the likelihood and confidence in finding high quality metabolite matches.

## THE HMDB IS FAIR-COMPLIANT

The HMDB is FAIR compliant (40) and details regarding its ‘FAIRness’ are provided under the ‘About HMDB’ menu tab. To ensure findability, all entries in the HMDB have a unique and permanent 7-digit HMDB identifier. To ensure accessibility, the HMDB website is open and free and its data download operation is compatible with all modern web browsers. The HMDB’s downloadable spectral data files are available in the universally readable nmrML (41) and mzML (42) formats. Furthermore, all MS/MS and GC–MS spectra are assigned SPLASH keys (43) for rapid spectral querying and matching. Likewise, all the HMDB’s chemical structures are accessible in canonical SMILES, SDF, MOL, PDB, InChI and InChIKey formats, while all sequence (DNA and protein) data are stored in FASTA format. To ensure interoperability, all textual data and metadata in the HMDB are written in English, all spectral data are in the mzML or nmrML exchange format, all chemicals are in canonical SMILES, SDF, MOL, PDB, InChI and InChIKey formats, all sequence (DNA and protein) data are stored in FASTA, all images are stored in PNG format, and all nomenclature for compounds and spectral data follows standard ontologies or vocabularies used to describe these entities. An extensive and well-annotated data download section is also provided with files available in standard TXT, CSV, JSON and XML formats. To ensure re-usability, all the data in the HMDB is extensively sourced with clear information on provenance. The data in the HMDB are released under a Creative Commons Attribution-NonCommercial 4.0 International License.

## CONCLUSION AND FUTURE DIRECTIONS

The HMDB has grown considerably, both in size and scope, over the past 15 years. In 2007 it was a rather modest database with just 2180 molecules and very limited content, searching or visualization capabilities. Today, the HMDB is ∼100× larger (in terms of metabolite coverage) and more than 1000X larger in terms of data size and content. The HMDB now includes extensive spectral data, pathway data, physiological and disease data and it offers many different kinds of advanced visualization tools, search tools and download or data accessibility options. The enormous size and scope of the HMDB has also meant that it is becoming increasingly more challenging to update. In particular, the focus for this year's update (namely expanding compound and spectral or ‘observable’ coverage) precluded further expansion or updates with several other popular HMDB data collections (such as metabolite-disease, metabolite-gene or metabolite-SNP associations). Likewise, with the explosion in published metabolomics articles (now averaging > 2000 papers/year), it has become increasingly difficult to stay current with the literature on metabolite-biofluid associations and metabolite biomarker identifications. To address these challenges, more and more of the HMDB database updating process will rely on computationally based data harvesting and natural language processing techniques. These approaches are already being developed and trialled by the HMDB curation team through the ChemFOnt project. With ongoing improvements in deep learning methods for text analysis and other tools for text-to-data conversion, we are hopeful that these data harvesting and data updating approaches will allow previously neglected aspects of the HMDB to grow robustly and remain both current and relevant to the metabolomics community for many years to come.

In terms of future directions for the HMDB, we are particularly encouraged by ongoing developments in the area of *in silico* metabolomics. Significant improvements in the prediction accuracy (via quantum mechanics, machine learning or hybrid approaches) of many chemical or spectral observables (MS spectra, NMR spectra, IR spectra, RIs, CCS, etc.) along with important enhancements to scoring protocols suggest that *in silico* metabolomics will be a worthwhile direction for the HMDB to continue to pursue. As a result, HMDB users should expect to see even more (and more accurately) predicted observables and more sophisticated searches (such as neutral loss searching) being added. These should allow users to more accurately identify or annotate metabolites.

Beginning in 2022, the HMDB will also branch out with the introduction of a new sister database, called ‘HypoMet’, containing millions of hypothetical, biologically feasible metabolites generated computationally via BioTransformer (44) and a deep generative model called DeepMet. The data in HypoMet will also be enriched with various predicted spectral properties or predicted observables. The intent of HypoMet is to allow metabolomics researchers the opportunity to identify novel metabolites or generate testable hypotheses regarding the identity of hitherto unknown metabolites.

Another major effort with the HMDB over the coming two to three years will be the updating or supplementation of every HMDB entry with at least one machine-readable pathway diagram. This process is ∼60% complete and is expected to pick up speed in the coming year. The intent of this pathway updating process is to complement the work with the ChemFOnt project, allowing much more sophisticated interpretation (at a system-wide level) and integration of metabolomic, proteomic and/or genomic data. This work will see a tighter coupling between the analytical and statistical tools offered by MetaboAnalyst (45) and the visualization and query functions offered by HMDB.

## DATA AVAILABILITY

All data is freely available from the HMDB website or from the HMDB download page.
